# Towers, mad families, and unboundedness

**DOI:** 10.1007/s00153-023-00861-x

**Published:** 2023-02-12

**Authors:** Vera Fischer, Marlene Koelbing, Wolfgang Wohofsky

**Affiliations:** Institute of Mathematics, University of Vienna, Kolingasse 14–16, 1090 Wien, Austria

**Keywords:** Towers, Maximal almost disjoint families, Unboundedness, Canjar filters, Forcing, 03E35, 03E17

## Abstract

We show that Hechler’s forcings for adding a tower and for adding a mad family can be represented as finite support iterations of Mathias forcings with respect to filters and that these filters are ${\mathcal {B}}$-Canjar for any countably directed unbounded family ${\mathcal {B}}$ of the ground model. In particular, they preserve the unboundedness of any unbounded scale of the ground model. Moreover, we show that ${\mathfrak {b}}=\omega _1$ in every extension by the above forcing notions.

## Introduction

In this paper, we analyze Hechler’s forcings from [7] for adding a tower (see Sect. 3) and for adding a mad family (see Sect. 4), after giving some preliminaries on ${\mathcal {B}}$-Canjar filters in Sect. 2.

The forcings consist of finite conditions approximating a generic tower or a generic mad family, respectively. We first show that the poset for adding a tower can be represented as a finite support iteration, where each iterand adds a single real to the tower (which diagonalizes the initial part of the tower). In fact, each such iterand is equivalent to Mathias forcing with respect to the filter generated by the respective initial part of the tower (see Lemma 3.5). For the forcing adding a mad family, the situation is analogous (see Lemma 4.5); in this case, the filter is generated by the complements of the elements of the initial part of the mad family. It follows from these representations that the above forcing notions are $\sigma$-centered (see Corollary 3.6 and Corollary 4.6) in many cases of interest.

The main results of this paper show that the above posets preserve the unboundedness of any countably directed unbounded family of the ground model (see Theorem 3.7 and Theorem 4.7); in particular, any unbounded ground model scale is preserved. We actually prove that, for a given countably directed unbounded family ${\mathcal {B}}$ of the ground model, all the filters which are involved in the representation of Hechler’s posets are ${\mathcal {B}}$-Canjar, i.e., the corresponding Mathias forcings preserve the unboundedness of ${\mathcal {B}}$. To verify ${\mathcal {B}}$-Canjarness, we use a combinatorial characterization from [5] (see Theorem 2.3), together with a genericity argument. In Sect. 5, we conclude that ${\mathfrak {b}}=\omega _1$ holds true in every extension by one of Hechler’s forcings, using that they can be decomposed into a forcing which adds an unbounded family of size $\omega _1$ and a forcing which preserves the unboundedness of this family (see Corollaries 5.1 and 5.2). Finally, in Sect. 6, we list some open questions.

In [3], the authors of this paper define a forcing which adds a refining matrix of regular height $\lambda$, i.e., a refining system of mad families of height $\lambda$ without common refinement. There is always a refining matrix of height ${\mathfrak {h}}$ (which is the minimal possible height), where ${\mathfrak {h}}$ is the well-known distributivity number. In order to get a model with a refining matrix of regular height $\lambda > {\mathfrak {h}}$, it is shown that the forcing to add the refining matrix keeps the bounding number ${\mathfrak {b}}$ (and hence ${\mathfrak {h}}$) small: to this end, the forcing is represented as an iteration of Mathias forcings with respect to filters, which are shown to be ${\mathcal {B}}$-Canjar, where ${\mathcal {B}}$ is the family of ground model reals; this ensures that ${\mathcal {B}}$ is unbounded in the final model, witnessing that ${\mathfrak {b}}$ is small.

Since a refining matrix consists of mad families as well as (along the branches of its corresponding tree) towers, the forcing used in [3] is an elaborate combination of Hechler’s poset for adding a mad family and a tower, respectively. The proof that the forcing from [3] preserves the unboundedness of the ground model reals is a more complicated version of the proofs given in this paper.

## ${\mathcal {B}}$-Canjar filters

In this section, we will give the necessary preliminaries about ${\mathcal {B}}$-Canjar filters and the preservation of unboundedness.

### Definition 2.1

Let ${\mathcal {F}}\subseteq {\mathcal {P}}(\omega )$ be a filter containing the Fréchet filter. *Mathias forcing with respect to* ${\mathcal {F}}$ (denoted by ${\mathbb {M}}({\mathcal {F}})$) is the set of pairs (*s*, *A*) with $s\in 2^{<\omega }$ and $A\in {\mathcal {F}}$, where the order is defined as follows: $(t,B)\le (s,A)$ if

1. $$t\unrhd s$$, i.e., *t* extends *s*,
2. $$B\subseteq A$$,
3. for each $n\ge |s|$, if $t(n)=1$, then $n\in A$.

Note that ${\mathbb {M}}(\mathcal {F})$ is $\sigma$-centered: for $s \in 2^{<\omega }$, the set $\{ (s,A) \mid A \in \mathcal {F}\}$ is clearly centered (i.e., finitely many conditions have a common lower bound). Also note that Mathias forcing with respect to a countably generated filter has a countable dense subset, and therefore is forcing equivalent to Cohen forcing ${\mathbb {C}}$. For $f,g\in \omega ^\omega$, we write $f\le ^*g$ if $f(n)\le g(n)$ for all but finitely many $n\in \omega$. We say that ${\mathcal {B}}\subseteq \omega ^\omega$ is an *unbounded family*, if there exists no $g\in \omega ^\omega$ with $f\le ^* g$ for all $f\in {\mathcal {B}}$. The *bounding number* ${\mathfrak {b}}$ is the smallest size of an unbounded family in $\omega ^\omega$. A family ${\mathcal {B}}\subseteq \omega ^\omega$ is called *countably directed* if the following closure property holds:

$$\begin{aligned} \forall {\mathcal {A}} \subseteq {\mathcal {B}}\; (|{\mathcal {A}}| = \aleph _0 \rightarrow \exists f \in {\mathcal {B}}\; \forall g \in {\mathcal {A}} \; g \le ^* f). \end{aligned}$$

A filter $\mathcal {F}$ is *Canjar* if ${\mathbb {M}}(\mathcal {F})$ does not add a dominating real over the ground model (i.e., the ground model reals remain unbounded). We are interested in the following generalization of Canjarness:

### Definition 2.2

Let ${\mathcal {B}}\subseteq \omega ^\omega$ be an unbounded family. A filter $\mathcal {F}$ on $\omega$ is ${\mathcal {B}}$-*Canjar* if ${\mathbb {M}}(\mathcal {F})$ preserves the unboundedness of ${\mathcal {B}}$ (i.e., ${\mathcal {B}}$ is still unbounded in the extension by ${\mathbb {M}}(\mathcal {F})$).

### A combinatorial characterization of ${\mathcal {B}}$-Canjarness

Later, we will prove that certain filters are ${\mathcal {B}}$-Canjar. A combinatorial characterization of Canjarness has been given by Hrušák-Minami [8], which has been generalized to ${\mathcal {B}}$-Canjarness for well-ordered unbounded families ${\mathcal {B}}$ by Guzmán-Hrušák-Martínez [4]. This has been extended to countably directed unbounded families by Guzmán-Kalajdzievski [5].

Let $\mathcal {F}$ be a filter on $\omega$; recall that a set $X \subseteq [\omega ]^{<\omega }$ is in $({\mathcal {F}}^{<\omega })^+$ if and only if for each $A \in \mathcal {F}$ there is an $s \in X$ with $s \subseteq A$. Note that if ${\mathcal {G}}\subseteq \mathcal {F}$ are filters and $X\in ({\mathcal {F}}^{<\omega })^+$, then $X\in ({{\mathcal {G}}}^{<\omega })^+$.

Given ${\bar{X}} = \langle X_n \mid n \in \omega \rangle$ (with $X_n \subseteq [\omega ]^{<\omega }$ for each $n \in \omega$), and $f \in \omega ^\omega$, let

$$\begin{aligned} {\bar{X}}_f = \bigcup _{n \in \omega } (X_n \cap {\mathcal {P}}(f(n))). \end{aligned}$$

#### Theorem 2.3

Let ${\mathcal {B}}\subseteq \omega ^\omega$ be a countably directed unbounded family. A filter ${\mathcal {F}}$ on $\omega$ is ${\mathcal {B}}$-Canjar if and only if the following holds: for each sequence ${\bar{X}} = \langle X_n \mid n \in \omega \rangle \subseteq ({\mathcal {F}}^{<\omega })^+$, there exists an $f \in {\mathcal {B}}$ such that ${\bar{X}}_f \in ({\mathcal {F}}^{<\omega })^+$.

#### Proof

See [5, Proposition 10]. $$\square $$

It is well-known that Cohen forcing ${\mathbb {C}}$ preserves^1^ the unboundedness of every unbounded family. As mentioned above, Mathias forcing with respect to a countably generated filter is forcing equivalent to ${\mathbb {C}}$, and hence any countably generated filter is ${\mathcal {B}}$-Canjar for every unbounded family ${\mathcal {B}}$. To illustrate the characterization of ${\mathcal {B}}$-Canjarness from Theorem 2.3, we want to provide the following easy combinatorial proof:

#### Lemma 2.4

Let ${\mathcal {B}}$ be a countably directed unbounded family. Then every countably generated filter is ${\mathcal {B}}$-Canjar.

#### Proof

Let $\mathcal {F}$ be a filter generated by $\{ a_n \mid n<\omega \}$, i.e., $A \in \mathcal {F}$ if and only if $a_n \subseteq A$ for some $n \in \omega$. Let ${\bar{X}}=\langle X_n \mid n\in \omega \rangle \subseteq (\mathcal {F}^{<\omega })^+$. For every $n\in \omega$, let $s_n\in X_n$ with $s_n\subseteq \bigcap _{k<n} a_k$ (such $s_n$ exists since $X_n \in ({\mathcal {F}}^{<\omega })^+$). Let $g\in \omega ^\omega$ be such that $g(n) = \max (s_n)$ for every $n\in \omega$. Since ${\mathcal {B}}$ is unbounded, we can pick $f\in {\mathcal {B}}$ such that $f(n)>g(n)$ for infinitely many *n*. It is easy to check that $s_n\in {\bar{X}}_f$ for infinitely many *n*, and this implies that ${\bar{X}}_f\in (\mathcal {F}^{<\omega })^+$, as desired. $\square$

Later, we will actually use the following lemma (which is again based on the characterization from the above Theorem 2.3) to show that a filter is ${\mathcal {B}}$-Canjar.

#### Lemma 2.5

Let $V \subseteq W$ be models of ZFC, and assume that ${\mathcal {B}}\subseteq \omega ^\omega \cap V$ is unbounded and countably directed in *W*, and that $\mathcal {F}\in W$ is a filter on $\omega$. Moreover, assume the following: for each sequence $\langle X_n \mid n \in \omega \rangle \subseteq ({\mathcal {F}}^{<\omega })^+$ there exists a sequence $\langle s_n \mid n \in \omega \rangle$, as well as a model $V'$ with $V \subseteq V' \subseteq W$ such that

1. $$\langle s_n \mid n \in \omega \rangle \in V'$$,
2. $$s_n \in X_n$$ for each $$n \in \omega $$,
3. for each $D \in [\omega ]^\omega \cap V'$ and for each $A \in \mathcal {F}$, there exists $n \in D$ such that $s_n \subseteq A$.

Then $\mathcal {F}$ is ${\mathcal {B}}$-Canjar (in *W*).

#### Proof

We want to show that $\mathcal {F}$ is ${\mathcal {B}}$-Canjar by proving its characterization given by Theorem 2.3. So suppose a sequence $\langle X_n \mid n \in \omega \rangle \subseteq ({\mathcal {F}}^{<\omega })^+$ is given. By the hypothesis of the lemma, we can fix $\langle s_n \mid n \in \omega \rangle$ and $V'$ satisfying (1)–(3). Due to (1), there is $g \in V'$ such that $s_n \subseteq g(n)$ for each $n \in \omega$. Since ${\mathcal {B}}$ is unbounded in *W*, there is an $f \in {\mathcal {B}}$ such that $g \not \ge ^*f$ (i.e., $g(n) < f(n)$ for infinitely many $n \in \omega$); to finish the proof, we want to show that

$$\begin{aligned} {\bar{X}}_f = \bigcup _{n \in \omega } (X_n \cap {\mathcal {P}}(f(n))) \end{aligned}$$

is in $({\mathcal {F}}^{<\omega })^+$. So fix $A \in \mathcal {F}$. We will find $s \in {\bar{X}}_f$ with $s \subseteq A$. Note that both *f* (which is actually in *V*) and *g* are in $V'$, so there is an infinite set $D \in V'$ such that $g(n) \le f(n)$ for each $n \in D$. Now use (3) to obtain an $n \in D$ with $s_n \subseteq A$; observe that $s_n \in X_n$ by (2), and $s_n \subseteq g(n) \le f(n)$, hence $s_n \in {\bar{X}}_f$, as desired. $\square$

### Preservation of unboundedness at limits

We will also use the following theorem by^2^ Judah-Shelah [9] about preservation of unboundedness in finite support iterations:

#### Theorem 2.6

Suppose $\{ {{\,\mathrm{{\mathbb {P}}}\,}}_\alpha , \dot{{\mathbb {Q}}}_\alpha \mid \alpha <\delta \}$ is a finite support iteration of c.c.c. partial orders of limit length $\delta$, and ${\mathcal {B}}\subseteq \omega ^\omega$ is unbounded and countably directed. Moreover, suppose thatThen ${{\,\mathrm{\Vdash }\,}}_{{{\,\mathrm{{\mathbb {P}}}\,}}_\delta }$ “${\mathcal {B}}$ is an unbounded family”.

#### Proof

See [2, Theorem 3.5.2]. $$\square $$

## Hechler’s tower forcing

In this section, we analyze Hechler’s forcing from [7] to add a tower. First, we give some basic definitions: For $a, b \in [\omega ]^\omega$, we say that $b \subseteq ^* a$ if $b \setminus a$ is finite, i.e., $\subseteq ^*$ denotes almost-inclusion. For a sequence $\langle a_\xi \mid \xi < \delta \rangle \subseteq [\omega ]^\omega$, we say that $b \in [\omega ]^\omega$ is a *pseudo-intersection* of $\langle a_\xi \mid \xi < \delta \rangle$ if $b \subseteq ^* a_\xi$ for each $\xi < \delta$. We say that $\langle a_\xi \mid \xi < \delta \rangle$ is a *tower of length* $\delta$ if $a_\eta \subseteq ^* a_\xi$ for any $\eta > \xi$, and it does not have an infinite pseudo-intersection. The *tower number* ${\mathfrak {t}}$ is the smallest length of a tower.

The definition of the forcing we are giving here is not exactly as in [7], but it is easy to see that it is equivalent. Let $\lambda$ be a regular uncountable cardinal.

### Definition 3.1

$\mathbb {TOW}_\lambda$ is defined as follows: $p \in \mathbb {TOW}_\lambda$ if *p* is a function with finite domain, $\textrm{dom}(p) \subseteq \lambda$, and for each $\alpha \in \textrm{dom}(p)$, we have

$$\begin{aligned} p(\alpha ) = (s^p_\alpha , f^p_\alpha ) = (s_\alpha , f_\alpha ), \end{aligned}$$

where

1. $$s_\alpha \in 2^{<\omega }$$,
2. for each $\beta \in \textrm{dom}(p)$ with $\beta <\alpha$, $|s_\beta | \ge |s_\alpha |$,
3. $$\textrm{dom}(f_\alpha ) \subseteq \textrm{dom}(p) \cap \alpha $$,
4. $$f_\alpha : \textrm{dom}(f_\alpha ) \rightarrow \omega $$,
5. whenever $\beta \in \textrm{dom}(f_\alpha )$, and $n \in \omega$ with $n \in \textrm{dom}(s_\beta ) \cap \textrm{dom}(s_\alpha )$ and $n \ge f_\alpha (\beta )$, we have
  $$\begin{aligned} s_\beta (n) = 0 \rightarrow s_\alpha (n) = 0. \end{aligned}$$

The order on $\mathbb {TOW}_\lambda$ is defined as follows: $q \le p$ (“*q* is stronger than *p*”) if

1. $$\textrm{dom}(p) \subseteq \textrm{dom}(q)$$,
2. and for each $\alpha \in \textrm{dom}(p)$, we have
  - $$s^p_\alpha \unlhd s^q_\alpha $$,
  - $\textrm{dom}(f^p_\alpha ) \subseteq \textrm{dom}(f^q_\alpha )$ and $f^p_\alpha (\beta ) \ge f^q_\alpha (\beta )$ for each $\beta \in \textrm{dom}(f^p_\alpha )$.

Given a generic filter *G* for $\mathbb {TOW}_\lambda$, we define, for each $\alpha < \lambda$,

$$\begin{aligned} a_\alpha := \bigcup \{ s^p_\alpha \mid p \in G \wedge \alpha \in \textrm{dom}(p) \}. \end{aligned}$$

It is not difficult to verify that the generic object $\langle a_\alpha \mid \alpha <\lambda \rangle$ added by $\mathbb {TOW}_\lambda$ is a tower of length $\lambda$.

### Complete subforcings

We will now show that Hechler’s forcing for adding a tower of length $\lambda$ has many complete subforcings. Let us start with a useful definition:

#### Definition 3.2

A condition $p\in \mathbb {TOW}_\lambda$ is called *full* if there exists an $N\in \omega$ such that for all $\alpha \in \textrm{dom}(p)$

1. $$ |s_\alpha ^p|=N$$,
2. $$N \ge \max (\textrm{rng}(f_\alpha ^p) )$$,
3. $\forall \beta \in \textrm{dom}(p)$ with $\beta <\alpha$ it holds that $\beta \in \textrm{dom}(f_{\alpha }^p)$.

The set of full conditions is dense:

#### Lemma 3.3

For every condition $p\in \mathbb {TOW}_\lambda$ there exists a full condition *q* with $q\le p$ and $\textrm{dom}(q)=\textrm{dom}(p)$. In particular the set of full conditions is dense in $\mathbb {TOW}_\lambda$.

#### Proof

First extend *p* by defining $f^p_\alpha (\beta ):=|s_\alpha ^p|$ for every $\alpha ,\beta \in \textrm{dom}(p)$ with $\beta <\alpha$ for which $f^p_\alpha (\beta )$ was not defined before. It is easy to see that this extension yields a condition which fulfills (3). Now let $N \ge \max (\textrm{rng}(f_\alpha ^p) ), |s_\alpha ^p|$ for every $\alpha \in \textrm{dom}(p)$. For every $\beta \in \textrm{dom}(p)$ extend $s_\beta ^p$ with 0’s to length *N*. It is easy to see that this is a condition and it is full. $\square$

For any $C \subseteq \lambda$, let $\mathbb {TOW}_C = \{ p\in \mathbb {TOW}_\lambda \mid \textrm{dom}(p)\subseteq C \}$. In particular, for any $\alpha \le \lambda$, we have $\mathbb {TOW}_\alpha = \{ p\in \mathbb {TOW}_\lambda \mid \textrm{dom}(p)\subseteq \alpha \}$. Moreover, for $p\in \mathbb {TOW}_\lambda$, let $p\upharpoonright \!\upharpoonright C$ be the condition $p'$ with $\textrm{dom}(p')=\textrm{dom}(p)\cap C$, and $s_\alpha ^{p'}=s_\alpha ^{p}$, and $f_\alpha ^{p'}=f_\alpha ^{p}\upharpoonright C$ for each $\alpha \in \textrm{dom}(p')$. Clearly, $p\upharpoonright \!\upharpoonright C$ is a condition in $\mathbb {TOW}_C$. Note that if $C \subseteq \lambda$ is downward closed (i.e., if *C* is an ordinal), then $p\upharpoonright \!\upharpoonright C=p\upharpoonright C$.

#### Lemma 3.4

Let $C \subseteq \alpha \le \lambda$. Then $\mathbb {TOW}_C$ is a complete subforcing of $\mathbb {TOW}_\alpha$. Moreover, if $p\in \mathbb {TOW}_\alpha$ is a full condition, then $p\upharpoonright \!\upharpoonright C$ is a reduction of *p* to $\mathbb {TOW}_C$.

In particular, $\mathbb {TOW}_\beta$ is a complete subforcing of $\mathbb {TOW}_\alpha$ for each $\beta < \alpha \le \lambda$, and, if $p\in \mathbb {TOW}_\alpha$ is a full condition (in this case, it is easy to see that it is actually not necessary to assume that *p* is full), then $p \upharpoonright \beta$ is a reduction of *p* to $\mathbb {TOW}_\beta$. In fact, this is all we are going to need in this paper. Nevertheless, we decided to prove the more general version (for sets *C* which are not an ordinal) because it might be useful for future applications.

#### Proof of Lemma 3.4

We first show that $\mathbb {TOW}_C \subseteq _{ic} \mathbb {TOW}_\alpha$, i.e., incompatible conditions from $\mathbb {TOW}_C$ are incompatible in $\mathbb {TOW}_\alpha$. Let $p_0, p_1\in \mathbb {TOW}_C$ and $q\in \mathbb {TOW}_{\alpha }$ with $q\le p_0, p_1$. We have to show that there exists a condition $q'\in \mathbb {TOW}_C$ with $q'\le p_0,p_1$. Let $q':= q\upharpoonright \!\upharpoonright C$. It is very easy to check that $q'$ is as we wanted.

Let $p\in \mathbb {TOW}_\alpha$. We want to define a reduction of *p* to $\mathbb {TOW}_C$. Let $p'\le p$ be a full condition (see Lemma 3.3), and let $N_{p'} \in \omega$ be such that $|s_\beta ^{p'}|=N_{p'}$ for all $\beta \in \textrm{dom}(p')$. Let . Let  with $q\in \mathbb {TOW}_C$; by appending 0’s if necessary we can assume that there is $N_{q} \in \omega$ such that $N_{q} \ge N_{p'}$ and $|s_\beta ^{q}|=N_{q}$ for all $\beta \in \textrm{dom}(q)$ (we do not need to assume that *q* is full). We have to show that *q* is compatible with *p*. To show this, we define a witness *r* as follows. Let $\textrm{dom}(r):= \textrm{dom}(p') \cup \textrm{dom}(q) = (\textrm{dom}(p') \setminus C) \,\,\dot{\cup }\,\, \textrm{dom}(q)$. For $\beta \in \textrm{dom}(q)\cap \textrm{dom}(p')$, let $\textrm{dom}(f_\beta ^r):= \textrm{dom}(f_\beta ^q)\cup \textrm{dom}(f_\beta ^{p'})$ and let $f_\beta ^r(\delta ) := f_\beta ^q(\delta )$ for every $\delta \in \textrm{dom}(f_\beta ^q)$ and $f_\beta ^r(\delta ) := f_\beta ^{p'}(\delta )$ for every $\delta \in \textrm{dom}(f_\beta ^{p'}) \setminus \textrm{dom}(f_\beta ^q)$, and for $\beta \in \textrm{dom}(q)\setminus \textrm{dom}(p')$, let $f_\beta ^r:= f_\beta ^q$. For $\beta \in \textrm{dom}(p')\setminus \textrm{dom}(q)$, let $f_\beta ^r:= f_\beta ^{p'}$. For $\beta \in \textrm{dom}(q)$, let $s_\beta ^r := s_\beta ^q$. For $\beta \in \textrm{dom}(p') \setminus C$, define $s_\beta ^r \unrhd s_\beta ^{p'}$ with $|s_\beta ^r| = N_q$ as follows: for each $n \in [N_{p'}, N_q)$, let $s_\beta ^r(n) = 1$ if and only if there exists $\delta > \beta$ such that $\delta \in \textrm{dom}(p') \cap C$ and $s_\delta ^q(n) = 1$. Note that $|s_\beta ^r| = N_q$ for each $\beta \in \textrm{dom}(r)$.

The only non-trivial part in showing that *r* is a condition in $\mathbb {TOW}_\alpha$ is verifying Definition 3.1(5). So assume that $\beta < \gamma$, $\beta \in \textrm{dom}(f_\gamma ^r)$, $n\ge f_\gamma ^r(\beta )$ and $s_\gamma ^r(n)=1$. We have to show that $s_{\beta }^r(n)=1$. In case both $\gamma$ and $\beta$ belong to $\textrm{dom}(q)$, this just follows from the fact that *q* is a condition; otherwise, both $\gamma$ and $\beta$ belong to $\textrm{dom}(p')$, and at least one of the two does not belong to *C*. If $n < N_{p'}$, we get that $s_{\beta }^r(n)=1$ by definition of *r* and the fact that $p'$ is a condition. So we can assume that $n \in [N_{p'}, N_q)$, and it remains to check the following three cases. *Case 1:* $\gamma \in \textrm{dom}(p') \cap C$ and $\beta \in \textrm{dom}(p') \setminus C$. Here, $s^r_\beta (n) = 1$ by definition: since $\gamma \in \textrm{dom}(p') \cap C \subseteq \textrm{dom}(q)$, we have $s^q_\gamma = s^r_\gamma$ by definition, and hence $s^q_\gamma (n) = 1$ by assumption; therefore, $s^r_\beta (n) = 1$ by definition of $s^r_\beta$. *Case 2:* $\gamma , \beta \in \textrm{dom}(p') \setminus C$. Since $s_\gamma ^r(n)=1$, by definition there exists $\delta > \gamma$ such that $\delta \in \textrm{dom}(p') \cap C \subseteq \textrm{dom}(q)$ and $s_\delta ^q(n) = 1$. Note that $\delta > \beta$, so by definition $s_\beta ^r(n) = 1$. *Case 3:* $\gamma \in \textrm{dom}(p') \setminus C$ and $\beta \in \textrm{dom}(p') \cap C$. Since $s_\gamma ^r(n)=1$, by definition there exists $\delta > \gamma$ such that $\delta \in \textrm{dom}(p') \cap C \subseteq \textrm{dom}(q)$ and $s_\delta ^q(n) = 1$. Recall that $p'$ is a full condition, so in particular $\beta \in \textrm{dom}(f^{p'}_\delta )$ and $f^{p'}_\delta (\beta ) \le N_{p'}$. Moreover, $q \le p'$, hence $\beta \in \textrm{dom}(f^q_\delta )$ and $f^q_\delta (\beta ) \le f^{p'}_\delta (\beta ) \le N_{p'}$. Therefore, due to $n \ge N_{p'} \ge f^q_\delta (\beta )$, it follows that $s^q_\beta (n) = 1$, and hence $s^r_\beta (n) = 1$ by definition of *r*. It is straightforward to check that $r \le q$ and $r \le p' \le p$. $\square$

### Iteration via filtered Mathias forcings

For $\alpha < \lambda$, $\mathbb {TOW}_\alpha$ is a complete subforcing of $\mathbb {TOW}_{\alpha +1}$ by Lemma 3.4, so we can form the quotient $\mathbb {TOW}_{\alpha +1}/\mathbb {TOW}_\alpha$. For a generic filter *G* for $\mathbb {TOW}_\alpha$, the quotient is defined by $\mathbb {TOW}_{\alpha +1}/\mathbb {TOW}_\alpha = \{ p\in \mathbb {TOW}_{\alpha +1} \mid \forall q\in G~ p\not \perp q\}$. Note that using Lemma 3.4 a (full) condition $p\in \mathbb {TOW}_{\alpha +1}$ belongs to $\mathbb {TOW}_{\alpha +1}/\mathbb {TOW}_\alpha$ if and only if $p\upharpoonright \alpha \in G$.

Moreover, because conditions in $\mathbb {TOW}_\lambda$ have finite domain,

$$\begin{aligned} \mathbb {TOW}_{\alpha } = \bigcup _{\delta < \alpha } \mathbb {TOW}_\delta \end{aligned}$$

for each limit ordinal $\alpha \le \lambda$; in other words, $\mathbb {TOW}_{\alpha }$ is the direct limit of the forcings $\mathbb {TOW}_\delta$ for $\delta < \alpha$. So $\mathbb {TOW}_\lambda$ is forcing equivalent to the finite support iteration of the quotients $\mathbb {TOW}_{\alpha +1}/\mathbb {TOW}_\alpha$ for $\alpha < \lambda$.

Recall that ${\mathbb {M}}({\mathcal {F}})$ denotes Mathias forcing with respect to the filter ${\mathcal {F}}$ (see Definition 2.1). We are now going to show that $\mathbb {TOW}_{\alpha +1}/\mathbb {TOW}_\alpha$ is forcing equivalent to ${\mathbb {M}}(\mathcal {F}_\alpha )$ for a filter $\mathcal {F}_\alpha$. Work in an extension by $\mathbb {TOW}_\alpha$, and note that, for each $\beta < \alpha$, a set $a_\beta$ has been added by $\mathbb {TOW}_\alpha$. Let

$$\begin{aligned} \mathcal {F}_\alpha := \langle \{ a_\beta \mid \beta < \alpha \} \rangle _{Fr\acute{e}ch{e}t}, \end{aligned}$$

i.e., $\mathcal {F}_\alpha$ is the filter generated by (the Fréchet filter and) the $\subseteq ^*$-decreasing sequence $\{a_\beta \mid \beta < \alpha \}$ added by $\mathbb {TOW}_{\alpha }$. Note that each element of $\mathcal {F}_\alpha$ is a superset of $a_\beta \setminus N$ for some $\beta <\alpha$ and $N\in \omega$.

The quotient $\mathbb {TOW}_{\alpha +1}/\mathbb {TOW}_\alpha$ adds the set $a_\alpha$. The following lemma will provide a dense embedding from $\mathbb {TOW}_{\alpha +1}/\mathbb {TOW}_\alpha$ to ${\mathbb {M}}(\mathcal {F}_\alpha )$ which preserves (the finite approximations of) the generic real $a_{\alpha }$. Therefore, $a_{\alpha }$ is also the generic real for ${\mathbb {M}}({\mathcal {F}_\alpha })$. Recall that the generic real for ${\mathbb {M}}({\mathcal {F}})$ is a pseudo-intersection of $\mathcal {F}$, and the definition of $\mathcal {F}_\alpha$ ensures that a pseudo-intersection of it is almost contained in $a_\beta$ for each $\beta < \alpha$, as it is the case for the real $a_\alpha$.

#### Lemma 3.5

$\mathbb {TOW}_{\alpha +1}/\mathbb {TOW}_{\alpha }$ is densely embeddable into ${\mathbb {M}}(\mathcal {F}_\alpha )$.

#### Proof

We work in a fixed extension by $\mathbb {TOW}_{\alpha }$ with generic filter *G*. The embedding $\iota$ is defined as follows: $\iota (p):= (s_{\alpha }^p,A)$ where

$$\begin{aligned} A:=\bigcap \limits _{\beta \in \textrm{dom}(f^p_{\alpha })}( a_\beta \cup f^p_{\alpha }(\beta ))\setminus |s_{\alpha }^p|. \end{aligned}$$

To see that it is a dense embedding, we have to check the following conditions:

1. (Density) For every condition $(s,A)\in {\mathbb {M}}(\mathcal {F}_{\alpha })$ there exists a condition *p* such that $\iota (p)\le (s,A)$.
2. (Incompatibility preserving) If *p* and $p'$ are incompatible, then so are $\iota (p)$ and $\iota (p')$.
3. (Order preserving) If $p'\le p$ then $\iota (p')\le \iota (p)$.

To show (1): Let $(s,A)\in {\mathbb {M}}(\mathcal {F}_\alpha )$. Since $A\in \mathcal {F}_\alpha$, there exist $\gamma <\alpha$ and $N\in \omega$ such that $a_\gamma \setminus N \subseteq A$. Extend *s* with 0’s to $s_{\alpha }$ such that $|s_{\alpha }|=\max (|s|,N)$ and let $\textrm{dom}(f_{\alpha }):=\{\gamma \}$ and $f_{\alpha }(\gamma ):=|s_{\alpha }|$. Let $p:=\{(\alpha ,(s_{\alpha },f_{\alpha }))\}\cup \{ (\gamma ,(a_\gamma \upharpoonright |s_\alpha |, \emptyset ))\}$. Note that *p* is full and $p\upharpoonright \alpha = \{ (\gamma ,(a_\gamma \upharpoonright |s_\alpha |, \emptyset ))\} \in G$, so $p \in \mathbb {TOW}_{\alpha +1}/G$. Now, $\iota (p)= (s_{\alpha },A')$ where

$$\begin{aligned} A'=\bigcap \limits _{\beta \in \textrm{dom}(f_{\alpha })}( a_\beta \cup f_{\alpha }(\beta ))\setminus |s_{\alpha }|. \end{aligned}$$

It follows that

$$\begin{aligned} A' = ( a_\gamma \cup f_{\alpha }(\gamma ))\setminus |s_{\alpha }| = a_\gamma \setminus |s_{\alpha }|\subseteq a_{\gamma }\setminus N \subseteq A. \end{aligned}$$

Therefore, and by the above, $s_{\alpha } \unrhd s$, $A'\subseteq A$, and $s_{\alpha }(n)=0$ for all $n\ge |s|$. So $\iota (p)=(s_{\alpha },A')\le (s,A)$.

We prove (2) by showing the contrapositive: Assume $\iota (p)=(s_\alpha ^p,A)$ and $\iota (p')=(s_\alpha ^{p'},A')$ are compatible. Define *q* as follows: $\textrm{dom}(q):= \textrm{dom}(p)\cup \textrm{dom}(p')$. For $\beta \in \textrm{dom}(q)$, let $\textrm{dom}(f_\beta ^q):= \textrm{dom}(f_\beta ^p)\cup \textrm{dom}(f_\beta ^{p'})$ and for $\rho \in \textrm{dom}(f_\beta ^q)$ let $f_\beta ^q(\rho )=\min (f_\beta ^p(\rho ),f_\beta ^{p'}(\rho ))$ (set $f_\beta ^p(\rho ) = \infty$, $f_\beta ^{p'}(\rho ) = \infty$ if not defined). Let $s_\alpha ^q:= s_\alpha ^p \cup s_\alpha ^{p'}$. Let $N \in \omega$ be such that $N \ge |s_\beta ^p|$ for each $\beta \in \textrm{dom}(p)$ and $N \ge |s_\beta ^{p'}|$ for each $\beta \in \textrm{dom}(p')$. Let $s_\beta ^q:=a_\beta \upharpoonright N$ for $\beta \in \textrm{dom}(q)$ with $\beta <\alpha$. The only non-trivial part in showing that *q* is a condition in $\mathbb {TOW}_{\alpha +1}/G$ is verifying Definition 3.1(5) for $\alpha$. We can assume without loss of generality that $s^p_\alpha \unlhd s^{p'}_\alpha = s^q_\alpha$. Let $\beta \in \textrm{dom}(f^q_\alpha )$, $n \ge f^q_\alpha (\beta )$, and $s^q_\alpha (n) = 1$. We have to show that $a_\beta (n) = 1$. In case $f^q_\alpha (\beta ) = f^{p'}_\alpha (\beta )$, we get that $a_\beta (n) = 1$ because $p'$ is a condition in the quotient. So let us assume that $f^q_\alpha (\beta ) = f^{p}_\alpha (\beta ) < f^{p'}_\alpha (\beta )$. If $n < |s^p_\alpha |$, then we are finished because *p* is a condition in the quotient. If $n \ge |s^p_\alpha |$, the compatibility of $(s_\alpha ^p,A)$ and $(s_\alpha ^{p'},A')$ implies $n \in A$. Since $(s_\alpha ^p,A) = \iota (p)$, the definition of $\iota$ in particular yields $n \in a_\beta \cup f^p_{\alpha }(\beta )$, so we are finished. It is straightforward to check that $q\le p,p'$.

To show (3): Let $p'\le p$. By definition that means: $s_{\alpha }^{p'} \unrhd s_{\alpha }^p$ and $\textrm{dom}(f_{\alpha }^{p'})\supseteq \textrm{dom}(f_{\alpha }^{p})$, and $f_{\alpha }^{p'}(\beta )\le f_{\alpha }^{p}(\beta )$ for $\beta \in \textrm{dom}(f_{\alpha }^{p})$; so

$$\begin{aligned} A':=\bigcap \limits _{\beta \in \textrm{dom}(f_{\alpha }^{p'})}( a_\beta \cup f_{\alpha }^{p'}(\beta ))\setminus |s_{\alpha }^{p'}| \subseteq \bigcap \limits _{\beta \in \textrm{dom}(f_{\alpha }^{p})}( a_\beta \cup f_{\alpha }^{p}(\beta ))\setminus |s_{\alpha }^{p}|=:A. \end{aligned}$$

Let $n\ge |s_{\alpha }^p|$ and $s_{\alpha }^{p'}(n)=1$. We have to show that $n\in A$; fix $\beta \in \textrm{dom}(f_{\alpha }^p)$ and show that $n\in a_\beta \cup f_{\alpha }^p(\beta )$. If $n<f_{\alpha }^p(\beta )$, this is clear. If $n\ge f_{\alpha }^p(\beta )$, it follows that $n\ge f_{\alpha }^{p'}(\beta )$, hence $s_\beta ^{p'}(n)=a_\beta (n)=1$, because $p'$ is a condition in the quotient. This shows that $\iota (p') = (s_{\alpha }^{p'},A') \le (s_{\alpha }^p,A) = \iota (p)$. $\square$

As a side result, let us mention that Hechler’s forcing for adding a tower is $\sigma$-centered:

#### Corollary 3.6

If $\lambda \le {\mathfrak {c}}$, then $\mathbb {TOW}_\lambda$ is $\sigma$-centered.

#### Proof

Since Mathias forcing with respect to a filter is always $\sigma$-centered (see the remark after Definition 2.1) and $\mathbb {TOW}_{\alpha +1}/\mathbb {TOW}_{\alpha }$ is densely embeddable into such a forcing by the above lemma, also $\mathbb {TOW}_{\alpha +1}/\mathbb {TOW}_{\alpha }$ is $\sigma$-centered.

So $\mathbb {TOW}_\lambda$ is a finite support iteration of $\sigma$-centered forcings of length at most ${\mathfrak {c}}$. As a matter of fact, the finite support iteration of $\sigma$-centered forcings of length strictly less than ${\mathfrak {c}}^+$ is $\sigma$-centered (the result was mentioned without proof in [10, proof of Lemma 2]; for a proof, see [1] or [6, Lemma 5.3.8]). $\square$

### The filters are ${\mathcal {B}}$-Canjar

Finally, we show that Hechler’s forcing $\mathbb {TOW}_\lambda$ preserves the unboundedness of countably directed unbounded families ${\mathcal {B}}$. More precisely, let *V* be the ground model over which we force with $\mathbb {TOW}_\lambda$, and let ${\mathcal {B}}\in V$ be a countably directed unbounded family of reals; we want to show that ${\mathcal {B}}$ is still unbounded in the extension by $\mathbb {TOW}_{\lambda }$. Since there always exists an unbounded family ${\mathcal {B}}$ of size ${\mathfrak {b}}$ which is countably directed, $\mathbb {TOW}_\lambda$ does not increase the bounding number ${\mathfrak {b}}$ (for more details, see Sect. 5; in fact, we argue there that we even get ${\mathfrak {b}}= \omega _1$ whenever we force with $\mathbb {TOW}_{\lambda }$).

In Sect. 3.2, we have defined filters $\mathcal {F}_\alpha$ for $\alpha < \lambda$ and have shown that $\mathbb {TOW}_\lambda$ is equivalent to the finite support iteration of the Mathias forcings ${\mathbb {M}}(\mathcal {F}_\alpha )$. So we can finish the proof by showing that the filters $\mathcal {F}_\alpha$ are ${\mathcal {B}}$-Canjar (and ${\mathbb {M}}(\mathcal {F}_\alpha )$ therefore preserves the unboundedness of ${\mathcal {B}}$), and using Theorem 2.6 at limits. In fact, we show the following:

#### Theorem 3.7

Let ${\mathcal {B}}$ be a countably directed unbounded family. Then $\mathbb {TOW}_\lambda$ preserves the unboundedness of ${\mathcal {B}}$. More precisely,

1. $\mathbb {TOW}_{\alpha }$ preserves the unboundedness of ${\mathcal {B}}$ for each $\alpha \le \lambda$,
2. $\mathcal {F}_\alpha$ is ${\mathcal {B}}$-Canjar for each $\alpha < \lambda$.

#### Proof

First note that ${\mathcal {B}}$ is countably directed in the extension by $\mathbb {TOW}_{\alpha }$ for each $\alpha \le \lambda$, since $\mathbb {TOW}_\alpha$ has the c.c.c. (and thus all countable sets of ground model objects are covered by a countable set of the ground model).

We prove (1) and (2) by (simultaneous) induction on $\alpha < \lambda$. Suppose (1) and (2) hold for all $\alpha ' < \alpha$.

**Proof of (1)**:

*In case* $\alpha = \alpha ' + 1$ *is a successor ordinal*, use the fact that (1) holds for $\alpha '$ by induction, so ${\mathcal {B}}$ is unbounded in the extension by $\mathbb {TOW}_{\alpha '}$; recall that, by Lemma 3.5, $\mathbb {TOW}_{\alpha } = \mathbb {TOW}_{\alpha '} * {\mathbb {M}}(\mathcal {F}_{\alpha '})$; since (2) holds for $\alpha '$ by induction, ${\mathbb {M}}(\mathcal {F}_{\alpha '})$ preserves the unboundedness of ${\mathcal {B}}$, hence the same is true for $\mathbb {TOW}_{\alpha }$, as desired.

*In case* $\alpha$ *is a limit ordinal*, we use the fact that $\mathbb {TOW}_{\alpha }$ is the finite support iteration of c.c.c. forcings, as well as that (1) holds for each $\alpha ' < \alpha$; so we can apply Theorem 2.6 to conclude (1) for $\alpha$.

**Proof of (2)**:

*In case* $\textrm{cf}(\alpha ) \le \omega$, just note that $\mathcal {F}_\alpha$ is countably generated; so, by Lemma 2.4, $\mathcal {F}_\alpha$ is ${\mathcal {B}}$-Canjar, as desired.

*In case* $\textrm{cf}(\alpha ) > \omega$, we proceed as follows (this is going to be the main technical part of the proof): in order to show that $\mathcal {F}_\alpha$ is ${\mathcal {B}}$-Canjar, it is sufficient to establish the hypothesis of Lemma 2.5.

Let *W* be the extension of *V* by $\mathbb {TOW}_\alpha$; note that $\mathcal {F}_\alpha$, which is generated by the Fréchet filter and $\{a_\beta \mid \beta < \alpha \}$, lies in *W*. Now observe that we have already proven (1) for $\alpha$ (without having used (2) for $\alpha$), i.e., we know that ${\mathcal {B}}$ is unbounded in *W*.

Now suppose that $\langle X_n \mid n \in \omega \rangle \subseteq ({\mathcal {F}_\alpha }^{<\omega })^+$ is given. We will find $\langle s_n \mid n \in \omega \rangle$ and $V'$ with $V \subseteq V' \subseteq W$ such that Lemma 2.5(1)–(3) hold. Since the $X_n$’s are essentially reals, the forcing $\mathbb {TOW}_\alpha$ has the c.c.c., and $\textrm{cf}(\alpha ) > \omega$, we can fix $\gamma < \alpha$ such that $\langle X_n \mid n \in \omega \rangle$ belongs to the extension of *V* by $\mathbb {TOW}_{\gamma }$; let $V'$ be the extension by $\mathbb {TOW}_{\gamma +1}$; clearly, $V \subseteq V' \subseteq W$.

For each $n \in \omega$, we have $a_\gamma \setminus n \in \mathcal {F}_\alpha$ and $X_n \in ({\mathcal {F}_\alpha }^{<\omega })^+$; therefore, for each *n*, there exists an $s \in X_n$ such that $s \subseteq a_\gamma \setminus n$. The same holds in $V'$ since $X_n\in V'$ for each *n* and $a_\gamma \in V'$. Since $\langle X_n \mid n\in \omega \rangle \in V'$, we can pick a sequence $\langle s_n \mid n\in \omega \rangle \in V'$ such that $s_n\in X_n$ and $s_n\subseteq a_\gamma \setminus n$ for every *n*.

It remains to show that Lemma 2.5(3) holds true. So fix $D \in [\omega ]^\omega \cap V'$; we have to prove that each element of $\mathcal {F}_\alpha$ contains (as a subset) an $s_n$ for some $n \in D$, i.e., that the following holds for each $\beta < \alpha$:

1 $$\begin{aligned} \forall k \in \omega \; \exists n \in D \;\; s_n \subseteq a_\beta \setminus k. \end{aligned}$$

*In case* $\beta \le \gamma$, this is easy: fix $k \in \omega$; recall that $a_\gamma \subseteq ^* a_\beta$, so we can pick $n \ge k$ with $n \in D$ such that $a_\gamma \setminus n \subseteq a_\beta$; but then $s_n \subseteq a_\gamma \setminus n \subseteq a_\beta \setminus k$, as desired.

*In case* $\beta > \gamma$, we show (1) by induction on $\beta$: assume we have shown it for every $\beta ' < \beta$; we will show it for $\beta$.

Fix $k \in \omega$, and work in the extension by $\mathbb {TOW}_\beta$ (note that *D* belongs to the extension by $\mathbb {TOW}_{\gamma +1}$, hence also to the extension by $\mathbb {TOW}_{\beta }$ due to $\beta \ge \gamma + 1$); observe that $a_\beta$ is added in the step from $\beta$ to $\beta + 1$, i.e., by the quotient forcing $\mathbb {TOW}_{\beta +1}/\mathbb {TOW}_\beta$ (which is equivalent to ${\mathbb {M}}(\mathcal {F}_\beta )$). We finish the proof by showing that the set

$$\begin{aligned} \{ q \in \mathbb {TOW}_{\beta +1}/\mathbb {TOW}_\beta \mid \exists n \in D \;\; q {{\,\mathrm{\Vdash }\,}}s_n \subseteq a_\beta \setminus k \} \end{aligned}$$

is dense. Let $p\in \mathbb {TOW}_{\beta +1}/\mathbb {TOW}_\beta$, so $p(\beta ) =: (s,f)$ where *f* is a function with $\textrm{dom}(f) \subseteq \beta$ finite. Let $\beta ' := \max (\textrm{dom}(f))$, and note that $\beta ' < \beta$. Moreover, let $\ell$ be large enough such that $a_{\beta '} \setminus \ell \subseteq a_{\beta ''}$ for each $\beta '' \in \textrm{dom}(f)$, and let $L := \max (\ell , k, |s|)$. Use (1) for $\beta '$ and *L* to pick $n \in D$ such that $s_n \subseteq a_{\beta '} \setminus L$; because $L \ge \ell$, it follows that $s_n \subseteq a_{\beta ''}\setminus L$ for each ${\beta ''} \in \textrm{dom}(f)$. Now strengthen *p* as follows. Extend *s* to $s^*$ in such a way that $s^*(m) = 1$ if $m \in s_n$ and $s^*(m) = 0$ if $m \notin s_n$ and $m \ge |s|$ (this is legitimate, because $s_n$ is a subset of each $a_{\beta ''}$ with ${\beta ''} \in \textrm{dom}(f)$); then it is easy to find a condition $q \in \mathbb {TOW}_{\beta +1}/\mathbb {TOW}_\beta$ such that $q\le p$ and $q(\beta ) = (s^*,f)$. Note that $q {{\,\mathrm{\Vdash }\,}}s_n \subseteq a_\beta \setminus k$, as desired. $\square$

## Hechler’s mad family forcing

In this section, we analyze Hechler’s forcing from [7] to add a mad family. Again, we start with some basic definitions: For $a, b \in [\omega ]^\omega$, we say that *a* and *b* are *almost disjoint* if $a \cap b$ is finite. Moreover, we say that $A \subseteq [\omega ]^\omega$ is an *almost disjoint family* if *a* and $a'$ are almost disjoint whenever $a, a' \in A$ with $a \ne a'$. An almost disjoint family *A* is *maximal* (called *mad family*) if for each $b \in [\omega ]^\omega$ there exists $a \in A$ such that $|b \cap a| = \aleph _0$. The *almost disjointness number* ${\mathfrak {a}}$ is the smallest size of an infinite mad family.

The definition of the forcing we are giving here is not exactly as in [7], but it is easy to see that it is equivalent. Let $\lambda$ be a regular uncountable cardinal.

### Definition 4.1

$\mathbb {MAD}_\lambda$ is defined as follows: $p \in \mathbb {MAD}_\lambda$ if *p* is a function with finite domain, $\textrm{dom}(p) \subseteq \lambda$, and for each $\alpha \in \textrm{dom}(p)$, we have $p(\alpha ) = (s^p_\alpha , h^p_\alpha ) = (s_\alpha , h_\alpha )$ where

1. $$s_\alpha \in 2^{<\omega }$$,
2. $$\textrm{dom}(h_\alpha ) \subseteq \textrm{dom}(p) \cap \alpha $$,
3. $$h_\alpha : \textrm{dom}(h_\alpha ) \rightarrow \omega $$,
4. whenever $\beta \in \textrm{dom}(h_\alpha )$, and $n \in \omega$ with $n \in \textrm{dom}(s_\beta ) \cap \textrm{dom}(s_\alpha )$ and $n \ge h_\alpha (\beta )$, we have
  $$\begin{aligned} s_\beta (n) = 0 \vee s_\alpha (n) = 0. \end{aligned}$$

The order on $\mathbb {MAD}_\lambda$ is defined as follows: $q \le p$ (“*q* is stronger than *p*”) if

1. $$\textrm{dom}(p) \subseteq \textrm{dom}(q)$$,
2. and for each $\alpha \in \textrm{dom}(p)$, we have
  - $$s^p_\alpha \unlhd s^q_\alpha $$,
  - $\textrm{dom}(h^p_\alpha ) \subseteq \textrm{dom}(h^q_\alpha )$ and $h^p_\alpha (\beta )\ge h^q_\alpha (\beta )$ for each $\beta \in \textrm{dom}(h^p_\alpha )$.

Given a generic filter *G* for $\mathbb {MAD}_\lambda$, we define, for each $\alpha < \lambda$,

$$\begin{aligned} a_\alpha := \bigcup \{ s^p_\alpha \mid p \in G \wedge \alpha \in \textrm{dom}(p) \}. \end{aligned}$$

It is not difficult to verify that the generic object $\{ a_\alpha \mid \alpha <\lambda \}$ added by $\mathbb {MAD}_\lambda$ is a mad family of size $\lambda$.

### Complete subforcings

We will now show that Hechler’s forcing for adding a mad family of size $\lambda$ has many complete subforcings. Let us start with a useful definition:

#### Definition 4.2

A condition $p\in \mathbb {MAD}_\lambda$ is called *full* if there exists an $N\in \omega$ such that for all $\alpha \in \textrm{dom}(p)$

1. $$ |s_\alpha ^p|=N$$,
2. $$N \ge \max (\textrm{rng}(h_\alpha ^p) )$$,
3. $\forall \beta \in \textrm{dom}(p)$ with $\beta <\alpha$ it holds that $\beta \in \textrm{dom}(h_{\alpha }^p)$.

The set of full conditions is dense:

#### Lemma 4.3

For every condition $p\in \mathbb {MAD}_\lambda$ there exists a full condition *q* with $q\le p$ and $\textrm{dom}(q)=\textrm{dom}(p)$. In particular the set of full conditions is dense in $\mathbb {MAD}_\lambda$.

#### Proof

First extend *p* by defining $h^p_\alpha (\beta ):=|s_\alpha ^p|$ for every $\alpha ,\beta \in \textrm{dom}(p)$ with $\beta <\alpha$ for which $h^p_\alpha (\beta )$ was not defined before. It is easy to see that this extension yields a condition which fulfills (3). Now let $N \ge \max (\textrm{rng}(h_\alpha ^p) ), |s_\alpha ^p|$ for every $\alpha \in \textrm{dom}(p)$. For every $\beta \in \textrm{dom}(p)$ extend $s_\beta ^p$ with 0’s to length *N*. It is easy to see that this is a condition and it is full. $\square$

For any $C \subseteq \lambda$, let $\mathbb {MAD}_C = \{ p\in \mathbb {MAD}_\lambda \mid \textrm{dom}(p)\subseteq C \}$. In particular, for any $\alpha \le \lambda$, we have $\mathbb {MAD}_\alpha = \{ p\in \mathbb {MAD}_\lambda \mid \textrm{dom}(p)\subseteq \alpha \}$. Moreover, for $p\in \mathbb {MAD}_\lambda$, let $p\upharpoonright \!\upharpoonright C$ be the condition $p'$ with $\textrm{dom}(p')=\textrm{dom}(p)\cap C$, and $s_\alpha ^{p'}=s_\alpha ^{p}$, and $h_\alpha ^{p'}=h_\alpha ^{p}\upharpoonright C$ for each $\alpha \in \textrm{dom}(p')$. Clearly, $p\upharpoonright \!\upharpoonright C$ is a condition in $\mathbb {MAD}_C$. Note that if $C \subseteq \lambda$ is downward closed (i.e., if *C* is an ordinal), then $p\upharpoonright \!\upharpoonright C=p\upharpoonright C$.

#### Lemma 4.4

Let $C \subseteq \alpha \le \lambda$. Then $\mathbb {MAD}_C$ is a complete subforcing of $\mathbb {MAD}_\alpha$. Moreover, if $p\in \mathbb {MAD}_\alpha$ is a full condition, then $p\upharpoonright \!\upharpoonright C$ is a reduction of *p* to $\mathbb {MAD}_C$.

Before proving the lemma, let us recall that in the context of Hechler’s forcing to add a tower, we only used a special instance of Lemma 3.4, namely that $\mathbb {TOW}_\beta$ is a complete subforcing of $\mathbb {TOW}_\alpha$, whereas here, we are going to use the more general version for sets $C\subseteq \alpha$ which are not ordinals. For Sect. 4.2, we need again only the special case of $\beta <\alpha$; the more general version is needed in Sect. 4.3. In Sect. 3.3, when dealing with $\mathbb {TOW}_\lambda$, we do not need such a more general version, for the following reason: the filter $\mathcal {F}_{\gamma +1}$ is always countably generated (just because $\{a_\gamma \setminus n \mid n \in \omega \}$ is a basis, due to the fact that $a_\gamma \subseteq ^* a_\beta$ for each $\beta < \gamma$), and so the analogue of the set $C \subseteq \alpha$ needed in Theorem 4.7 can be replaced by any upper bound which is a successor ordinal. This is not possible when dealing with $\mathbb {MAD}_\lambda$ since then $\mathcal {F}_\beta$ is never countably generated unless $\beta < \omega _1$.

#### Proof of Lemma 4.4

We first show that $\mathbb {MAD}_C\subseteq _{ic} \mathbb {MAD}_{\alpha }$, i.e., incompatible conditions from $\mathbb {MAD}_C$ are incompatible in $\mathbb {MAD}_\alpha$. Let $p_0, p_1\in \mathbb {MAD}_C$ and $q\in \mathbb {MAD}_{\alpha }$ with $q\le p_0, p_1$. We have to show that there exists a condition  with . Let . It is very easy to check that $q'$ is as we wanted.

Let $p\in \mathbb {MAD}_{\alpha }$. We want to define a reduction of *p* to $\mathbb {MAD}_C$. Let $p'\le p$ be a full condition (see Lemma 4.3), and let $N_{p'} \in \omega$ be such that $|s_\beta ^{p'}|=N_{p'}$ for all $\beta \in \textrm{dom}(p')$. Let . Let  with $q\in \mathbb {MAD}_C$. We have to show that *q* is compatible with *p*. To show this, we define a witness *r* as follows. Let $\textrm{dom}(r):= \textrm{dom}(p')\cup \textrm{dom}(q) = (\textrm{dom}(p') \setminus C) \,\,\dot{\cup }\,\, \textrm{dom}(q)$. For $\beta \in \textrm{dom}(q)$, let $s_\beta ^r:= s_\beta ^q$, and for $\beta \in \textrm{dom}(q)\cap \textrm{dom}(p')$, let $\textrm{dom}(h_\beta ^r):= \textrm{dom}(h_\beta ^q)\cup \textrm{dom}(h_\beta ^{p'})$ and let $h_\beta ^r(\delta ) := h_\beta ^q(\delta )$ for every $\delta \in \textrm{dom}(h_\beta ^q)$ and $h_\beta ^r(\delta ) := h_\beta ^{p'}(\delta )$ for every $\delta \in \textrm{dom}(h_\beta ^{p'}) \setminus \textrm{dom}(h_\beta ^q)$, and for $\beta \in \textrm{dom}(q)\setminus \textrm{dom}(p')$, let $h_\beta ^r:= h_\beta ^q$. For $\beta \in \textrm{dom}(p')\setminus \textrm{dom}(q)$, let $s_\beta ^r:= s_\beta ^{p'}$ and $h_\beta ^r:= h_\beta ^{p'}$.

The only non-trivial part in showing that *r* is a condition in $\mathbb {MAD}_\alpha$ is verifying Definition 4.1(4). So assume that $\beta < \gamma$, $\beta \in \textrm{dom}(h_\gamma ^r)$, $n \ge h_\gamma ^r(\beta )$ and $s_\gamma ^r(n) = 1$. We have to show that $s_{\beta }^r(n) = 0$ if it is defined. In case both $\gamma$ and $\beta$ belong to $\textrm{dom}(q)$, this just follows from the fact that *q* is a condition; otherwise, both $\gamma$ and $\beta$ belong to $\textrm{dom}(p')$, and at least one of the two does not belong to *C*. If $n < N_{p'}$, we get that $s_{\beta }^r(n) = 0$ by definition of *r* and the fact that $p'$ is a condition. But if $n \ge N_{p'}$, then either $s_{\beta }^r(n)$ or $s_{\gamma }^r(n)$ is not defined, and there is nothing to show. It is straightforward to check that $r \le q$ and $r \le p' \le p$. $\square$

### Iteration via filtered Mathias forcings

For $\alpha < \lambda$, $\mathbb {MAD}_\alpha$ is a complete subforcing of $\mathbb {MAD}_{\alpha +1}$ by Lemma 4.4, so we can form the quotient $\mathbb {MAD}_{\alpha +1}/\mathbb {MAD}_\alpha$. For a generic filter *G* for $\mathbb {MAD}_\alpha$, the quotient is defined by $\mathbb {MAD}_{\alpha +1}/\mathbb {MAD}_\alpha = \{ p\in \mathbb {MAD}_{\alpha +1} \mid \forall q\in G~ p\not \perp q\}$. Note that using Lemma 4.4 a full condition $p\in \mathbb {MAD}_{\alpha +1}$ belongs to $\mathbb {MAD}_{\alpha +1}/\mathbb {MAD}_\alpha$ if and only if $p\upharpoonright \alpha \in G$.

Moreover, because conditions in $\mathbb {MAD}_\lambda$ have finite domain,

$$\begin{aligned} \mathbb {MAD}_{\alpha } = \bigcup _{\delta < \alpha } \mathbb {MAD}_\delta \end{aligned}$$

for each limit ordinal $\alpha \le \lambda$; in other words, $\mathbb {MAD}_{\alpha }$ is the direct limit of the forcings $\mathbb {MAD}_\delta$ for $\delta < \alpha$. So $\mathbb {MAD}_\lambda$ is forcing equivalent to the finite support iteration of the quotients $\mathbb {MAD}_{\alpha +1}/\mathbb {MAD}_\alpha$ for $\alpha < \lambda$.

Recall that ${\mathbb {M}}({\mathcal {F}})$ denotes Mathias forcing with respect to the filter ${\mathcal {F}}$ (see Definition 2.1). We are now going to show that $\mathbb {MAD}_{\alpha +1}/\mathbb {MAD}_\alpha$ is forcing equivalent to ${\mathbb {M}}(\mathcal {F}_\alpha )$ for a filter $\mathcal {F}_\alpha$. Work in an extension by $\mathbb {MAD}_\alpha$, and note that, for each $\beta < \alpha$, a set $a_\beta$ has been added by $\mathbb {MAD}_\alpha$. Let

$$\begin{aligned} \mathcal {F}_\alpha := \langle \{ \omega \setminus a_\beta \mid \beta < \alpha \} \rangle _{Fr\acute{e}ch{e}t}, \end{aligned}$$

i.e., $\mathcal {F}_\alpha$ is the filter generated by (the Fréchet filter and) the complements of the members of the almost disjoint family $\{a_\beta \mid \beta < \alpha \}$ added by $\mathbb {MAD}_{\alpha }$.

The quotient $\mathbb {MAD}_{\alpha +1}/\mathbb {MAD}_\alpha$ adds the set $a_\alpha$. The following lemma will provide a dense embedding from $\mathbb {MAD}_{\alpha +1}/\mathbb {MAD}_\alpha$ to ${\mathbb {M}}(\mathcal {F}_\alpha )$ which preserves (the finite approximations of) the generic real $a_{\alpha }$. Therefore, $a_{\alpha }$ is also the generic real for ${\mathbb {M}}({\mathcal {F}_\alpha })$. Recall that the generic real for ${\mathbb {M}}({\mathcal {F}})$ is a pseudo-intersection of $\mathcal {F}$, and the definition of $\mathcal {F}_\alpha$ ensures that a pseudo-intersection of it is almost disjoint from $a_\beta$ for each $\beta < \alpha$, as it is the case for the real $a_\alpha$.

#### Lemma 4.5

$\mathbb {MAD}_{\alpha +1}/\mathbb {MAD}_{\alpha }$ is densely embeddable into ${\mathbb {M}}(\mathcal {F}_\alpha )$.

#### Proof

We work in a fixed extension by $\mathbb {MAD}_\alpha$. The embedding $\iota$ is defined as follows: $\iota (p):= (s_{\alpha }^p,A)$ where

$$\begin{aligned} A:= \bigcap \limits _{\beta \in \textrm{dom}(h_{\alpha }^p)}( (\omega \setminus a_\beta ) \cup h_{\alpha }^p(\beta ))\setminus |s_{\alpha }^p|. \end{aligned}$$

To see that it is a dense embedding, we have to check the following conditions:

1. (Density) For every condition $(s,A)\in {\mathbb {M}}(\mathcal {F}_\alpha )$ there exists a condition *p* such that $\iota (p)\le (s,A)$.
2. (Incompatibility preserving) If *p* and $p'$ are incompatible, then so are $\iota (p)$ and $\iota (p')$.
3. (Order preserving) If $p'\le p$ then $\iota (p')\le \iota (p)$.

To show (1): Let $(s,A)\in {\mathbb {M}}(\mathcal {F}_\alpha )$. Since $A\in \mathcal {F}_\alpha$, there exist finitely many $\{\beta _i\mid i<m\} \subseteq \alpha$ and $N\in \omega$ such that $\bigcap _{i<m} (\omega \setminus a_{\beta _i})\setminus N \subseteq A$. Extend *s* with 0’s to $s_{\alpha }$ such that $|s_{\alpha }|=\max (|s|,N )$ and define $h_\alpha$ by $\textrm{dom}(h_\alpha )=\{\beta _i\mid i<m\}$ and $h_\alpha (\beta _i)= |s_{\alpha }|$ for each $i<m$. Let $p:=\{(\alpha ,(s_{\alpha },h_{\alpha }))\}\cup \{ (\beta _i,(\langle \rangle , \emptyset ))\mid i<m \}$. Clearly, $p\upharpoonright \alpha$ belongs to any generic filter for $\mathbb {MAD}_\alpha$, and therefore $p\in \mathbb {MAD}_{\alpha +1}/\mathbb {MAD}_\alpha$. Now, $\iota (p)= (s_{\alpha },A')$ where

$$\begin{aligned} A'=\bigcap \limits _{\beta \in \textrm{dom}(h_{\alpha })}( (\omega \setminus a_\beta ) \cup h_{\alpha }(\beta ))\setminus |s_{\alpha }|. \end{aligned}$$

It follows that

$$\begin{aligned} A'= \bigcap _{\beta \in \textrm{dom}(h_{\alpha })}(\omega \setminus a_\beta )\setminus |s_{\alpha }|\subseteq \bigcap _{i<m}( \omega \setminus a_{\beta _i} )\setminus N \subseteq A. \end{aligned}$$

Therefore, and by the above, $s_{\alpha } \unrhd s$, $A'\subseteq A$, and $s_\alpha (n)=0$ for all $n\ge |s|$. So $\iota (p)=(s_{\alpha },A')\le (s,A)$.

We prove (2) by showing the contrapositive: Assume $\iota (p)=(s_\alpha ^p,A)$ and $\iota (p')=(s_\alpha ^{p'},A')$ are compatible. Define *q* as follows: $\textrm{dom}(q):= \textrm{dom}(p)\cup \textrm{dom}(p')$. For $\beta \in \textrm{dom}(q)$, let $s_\beta ^q:= s_\beta ^p\cup s_\beta ^{p'}$ (set $s_\beta ^p = \langle \rangle$, $s_\beta ^{p'}= \langle \rangle$ if not defined), $\textrm{dom}(h_\beta ^q):= \textrm{dom}(h_\beta ^p)\cup \textrm{dom}(h_\beta ^{p'})$ and for $\rho \in \textrm{dom}(h_\beta ^q)$ let $h_\beta ^q(\rho )=\min (h_\beta ^p(\rho ),h_\beta ^{p'}(\rho ))$ (set $h_\beta ^p(\rho ) = \infty$, $h_\beta ^{p'}(\rho ) = \infty$ if not defined). Similar to the proof of (2) in Lemma 3.5, it follows that *q* is a condition in the quotient and $q\le p,p'$.

To show (3): Let $p'\le p$. By definition that means: $s_{\alpha }^{p'} \unrhd s_{\alpha }^p$ and $\textrm{dom}(h_{\alpha }^{p'})\supseteq \textrm{dom}(h_{\alpha }^{p})$, and $h_{\alpha }^{p'}(\beta )\le h_{\alpha }^{p}(\beta )$ for $\beta \in \textrm{dom}(h_{\alpha }^{p})$; so

$$\begin{aligned} A':=\bigcap \limits _{\beta \in \textrm{dom}(h_{\alpha }^{p'})}( (\omega \setminus a_\beta ) \cup h_{\alpha }^{p'}(\beta ))\setminus |s_{\alpha }^{p'}| \subseteq \bigcap \limits _{\beta \in \textrm{dom}(h_{\alpha }^{p})}( (\omega \setminus a_\beta ) \cup h_{\alpha }^{p}(\beta ))\setminus |s_{\alpha }^{p}|=:A. \end{aligned}$$

Let $n\ge |s_{\alpha }^{p}|$ and $s_{\alpha }^{p'}(n)=1$. We have to show that $n\in A$; fix $\beta \in \textrm{dom}(h_{\alpha }^{p})$ and show that $n\in (\omega \setminus a_\beta ) \cup h_{\alpha }^{p}(\beta )$. If $n<h_{\alpha }^{p}(\beta )$, this is clear. If $n\ge h_{\alpha }^{p}(\beta )$, it follows that $n\ge h_{\alpha }^{p'}(\beta )$, hence $a_\beta (n)=0$, because $p'$ is a condition in the quotient. This shows that $\iota (p') = (s_{\alpha }^{p'},A') \le (s_{\alpha }^p,A) = \iota (p)$. $\square$

As a side result, let us mention that Hechler’s forcing for adding a mad family is $\sigma$-centered:

#### Corollary 4.6

If $\lambda \le {\mathfrak {c}}$, then $\mathbb {MAD}_\lambda$ is $\sigma$-centered.

#### Proof

The proof is completely analogous to the proof of Corollary 3.6. $\square$

### The filters are ${\mathcal {B}}$-Canjar

Finally, as we did in Sect. 3.3 for Hechler’s tower forcing $\mathbb {TOW}_\lambda$, we show that Hechler’s forcing $\mathbb {MAD}_\lambda$ preserves the unboundedness of countably directed unbounded families ${\mathcal {B}}$. More precisely, let *V* be the ground model over which we force with $\mathbb {MAD}_\lambda$, and let ${\mathcal {B}}\in V$ be a countably directed unbounded family of reals; we want to show that ${\mathcal {B}}$ is still unbounded in the extension by $\mathbb {MAD}_{\lambda }$. Since there always exists an unbounded family ${\mathcal {B}}$ of size ${\mathfrak {b}}$ which is countably directed, $\mathbb {MAD}_\lambda$ does not increase the bounding number ${\mathfrak {b}}$ (for more details, see Sect. 5; in fact, we argue there that we even get ${\mathfrak {b}}= \omega _1$ whenever we force with $\mathbb {MAD}_{\lambda }$).

In Sect. 4.2, we have defined filters $\mathcal {F}_\alpha$ for $\alpha < \lambda$ and have shown that $\mathbb {MAD}_\lambda$ is equivalent to the finite support iteration of the Mathias forcings ${\mathbb {M}}(\mathcal {F}_\alpha )$. So we can finish the proof by showing that the filters $\mathcal {F}_\alpha$ are ${\mathcal {B}}$-Canjar (and ${\mathbb {M}}(\mathcal {F}_\alpha )$ therefore preserves the unboundedness of ${\mathcal {B}}$), and using Theorem 2.6 at limits. In fact, we show the following:

#### Theorem 4.7

Let ${\mathcal {B}}$ be a countably directed unbounded family. Then $\mathbb {MAD}_\lambda$ preserves the unboundedness of ${\mathcal {B}}$. More precisely,

1. $\mathbb {MAD}_{\alpha }$ preserves the unboundedness of ${\mathcal {B}}$ for each $\alpha \le \lambda$,
2. $\mathcal {F}_\alpha$ is ${\mathcal {B}}$-Canjar for each $\alpha < \lambda$.

#### Proof

First note that ${\mathcal {B}}$ is countably directed in the extension by $\mathbb {MAD}_{\alpha }$ for each $\alpha \le \lambda$, since $\mathbb {MAD}_\alpha$ has the c.c.c. (and thus all countable sets of ground model objects are covered by a countable set of the ground model).

We prove (1) and (2) by (simultaneous) induction on $\alpha < \lambda$. Suppose (1) and (2) hold for all $\alpha ' < \alpha$.

**Proof of (1)**:

*In case* $\alpha = \alpha ' + 1$ *is a successor ordinal*, use the fact that (1) holds for $\alpha '$ by induction, so ${\mathcal {B}}$ is unbounded in the extension by $\mathbb {MAD}_{\alpha '}$; recall that, by Lemma 4.5, $\mathbb {MAD}_{\alpha } = \mathbb {MAD}_{\alpha '} * {\mathbb {M}}(\mathcal {F}_{\alpha '})$; since (2) holds for $\alpha '$ by induction, ${\mathbb {M}}(\mathcal {F}_{\alpha '})$ preserves the unboundedness of ${\mathcal {B}}$, hence the same is true for $\mathbb {MAD}_{\alpha }$, as desired.

*In case* $\alpha$ *is a limit ordinal*, we use the fact that $\mathbb {MAD}_{\alpha }$ is the finite support iteration of c.c.c. forcings, as well as that (1) holds for each $\alpha ' < \alpha$; so we can apply Theorem 2.6 to conclude (1) for $\alpha$.

**Proof of (2)**:

*In case* $\alpha < \omega _1$, just note that $\mathcal {F}_\alpha$ is countably generated; so, by Lemma 2.4, $\mathcal {F}_\alpha$ is ${\mathcal {B}}$-Canjar, as desired.

*In case* $\alpha \ge \omega _1$, we proceed as follows (this is going to be the main technical part of the proof): in order to show that $\mathcal {F}_\alpha$ is ${\mathcal {B}}$-Canjar, it is sufficient to establish the hypothesis of Lemma 2.5.

Let *W* be the extension of *V* by $\mathbb {MAD}_\alpha$; note that $\mathcal {F}_\alpha$, which is generated by the Fréchet filter and $\{ \omega \setminus {a_\beta } \mid \beta < \alpha \}$, lies in *W*. Now observe that we have already proven (1) for $\alpha$ (without having used (2) for $\alpha$), i.e., we know that ${\mathcal {B}}$ is unbounded in *W*.

Now suppose that $\langle X_n \mid n \in \omega \rangle \subseteq ({\mathcal {F}_\alpha }^{<\omega })^+$ is given. We will find $\langle s_n \mid n \in \omega \rangle$ and $V'$ with $V \subseteq V' \subseteq W$ such that Lemma 2.5(1)–(3) hold. Since the $X_n$’s are essentially reals and the forcing $\mathbb {MAD}_\alpha$ has the c.c.c., we can pick a countable “support” $C \subseteq \alpha$, i.e., a set *C* such that $\langle X_n \mid n \in \omega \rangle$ belongs to the extension by $\mathbb {MAD}_C$ (which is a complete subforcing of $\mathbb {MAD}_\alpha$ by Lemma 4.4); let $V'$ be the extension by $\mathbb {MAD}_C$; clearly, $V \subseteq V' \subseteq W$.

Enumerate *C* by $\{ \gamma _\ell \mid \ell < \omega \}$ and let $c^\ell := \omega \setminus {a_{\gamma _\ell }}$ for each $\ell \in \omega$. For each $n \in \omega$, we have $\bigcap _{\ell \le n} c^\ell \setminus n \in \mathcal {F}_\alpha$ and $X_n \in ({\mathcal {F}_\alpha }^{<\omega })^+$; therefore, for each *n*, there exists an $s \in X_n$ such that $s \subseteq \bigcap _{\ell \le n} c^\ell \setminus n$. The same holds in $V'$ since $X_n\in V'$ for each *n* and $c^\ell \in V'$ for each $\ell$. Since $\langle X_n \mid n\in \omega \rangle \in V'$ and $\langle c^\ell \mid \ell \in \omega \rangle \in V'$, we can pick a sequence $\langle s_n \mid n\in \omega \rangle \in V'$ such that $s_n\in X_n$ and $s_n\subseteq \bigcap _{\ell \le n} c^\ell \setminus n$ for every *n*.

It remains to show that Lemma 2.5(3) holds true. So fix $D \in [\omega ]^\omega \cap V'$; we have to prove that each element of $\mathcal {F}_\alpha$ contains (as a subset) an $s_n$ for some $n \in D$, i.e., that the following holds for each finite sequence $\langle \beta _i \mid i < N \rangle \subseteq \alpha$:

2 $$\begin{aligned} \forall k \in \omega \; \exists n \in D \;\; s_n \subseteq \bigcap \limits _{i < N} (\omega \setminus {a_{\beta _i}}) \setminus k. \end{aligned}$$

We first observe that (2) holds in case that $\{ \beta _i \mid i < N \} \subseteq C$: fix $k \in \omega$, and note that there is $m \in \omega$ such that for each $n \ge m$, we have

$$\begin{aligned} s_n \subseteq \bigcap \limits _{\ell \le n} c^\ell \setminus n \subseteq \bigcap \limits _{i < N} (\omega \setminus {a_{\beta _i}}) \setminus k, \end{aligned}$$

hence there is such an *n* in the infinite set *D*, as desired.

We now show (2) for arbitrary $\{ \beta _i \mid i < N \} \subseteq \alpha$, using a genericity argument. Let $N_C := \{ i \in N \mid \beta _i \in C \}$, and $N_{\alpha \setminus C} := \{ i \in N \mid \beta _i \notin C \}$, so $N = N_C \,\,\dot{\cup }\,\, N_{\alpha \setminus C}$.

Fix $k \in \omega$, and work in $V'$, the extension by $\mathbb {MAD}_{C}$ (note that $D \in V'$); observe that the $a_{\beta _i}$’s for $i \in N_{\alpha \setminus C}$ are added by the quotient forcing $\mathbb {MAD}_\alpha / \mathbb {MAD}_C$. We finish the proof by showing that the set

$$\begin{aligned} \{ q \in \mathbb {MAD}_\alpha / \mathbb {MAD}_C \mid \exists n \in D \;\; q {{\,\mathrm{\Vdash }\,}}s_n \subseteq \bigcap \limits _{i < N} (\omega \setminus {a_{\beta _i}}) \setminus k \} \end{aligned}$$

is dense. Let $p \in \mathbb {MAD}_\alpha / \mathbb {MAD}_C$; we can assume that $\beta _i \in \textrm{dom}(p)$ for each $i \in N$. For $i \in N_{\alpha \setminus C}$, let $p(\beta _i) =: (s^p_{\beta _i},h^p_{\beta _i})$. Let $L := \max (\{ k \} \cup \{|s^p_{\beta _i}| \mid i \in N_{\alpha \setminus C} \})$. Since (2) holds for $\beta _i$’s in *C* (as shown above), we can pick $n \in D$ such that

$$\begin{aligned} s_n \subseteq \bigcap \limits _{i \in N_C} (\omega \setminus {a_{\beta _i}}) \setminus L. \end{aligned}$$

Now extend *p* to *q* by extending all the $s^p_{\beta _i}$ with $i \in N_{\alpha \setminus C}$ with 0’s up to the maximum of $s_n$ (recall that we can always^3^ extend with 0’s, because this does not harm the requirement related to almost disjointness). So we get that *q* forces $m\in \omega \setminus {a_{\beta _i}}$ for all $i \in N_{\alpha \setminus C}$ and all $m\in s_n$, and hence

$$\begin{aligned} q {{\,\mathrm{\Vdash }\,}}s_n \subseteq \bigcap _{i \in N_{\alpha \setminus C}} (\omega \setminus {a_{\beta _i}}) \cap \bigcap _{i \in N_C} (\omega \setminus {a_{\beta _i}}) \setminus L \subseteq \bigcap _{i \in N} (\omega \setminus {a_{\beta _i}}) \setminus k, \end{aligned}$$

as desired. $$\square $$

## Conclusion

In this section, we present some facts about cardinal characteristics which easily follow from our analysis of $\mathbb {TOW}_\lambda$ and $\mathbb {MAD}_\lambda$.

First note that any unbounded scale, i.e., any unbounded set ${\mathcal {B}}=\{ f_i \mid i<\kappa \}$ such that $f_i\le ^* f_j$ for $i\le j$, is countably directed, because its length $\kappa$ has uncountable cofinality. Therefore, by Theorems 3.7 and 4.7, any unbounded scale of the ground model remains unbounded in the extension by $\mathbb {TOW}_\lambda$ and $\mathbb {MAD}_\lambda$, respectively. It is easy to see that there exists always an unbounded scale of length ${\mathfrak {b}}$. Assume $V\models ``{\mathfrak {b}}=\kappa$”. Then $V^{\mathbb {TOW}_\lambda }\models$ “there exists an unbounded scale of length $\kappa$ and there exists a tower of length $\lambda$”. In particular, this implies that $V^{\mathbb {TOW}_\lambda }\models ``{\mathfrak {b}}\le \kappa$”. The same argument works for $\mathbb {MAD}_\lambda$, therefore $V^{\mathbb {MAD}_\lambda }\models ``{\mathfrak {b}}\le \kappa$ and there exists an unbounded scale of length $\kappa$ and a mad family of size $\lambda$”.

Note that the above shows that ${\mathfrak {b}}= \omega _1$ holds in the extension by $\mathbb {TOW}_\lambda$ (or $\mathbb {MAD}_\lambda$) provided that ${\mathfrak {b}}= \omega _1$ holds in the ground model. But in fact the following argument shows that no assumption about ${\mathfrak {b}}$ in the ground model is necessary for this conclusion. The forcing $\mathbb {TOW}_\lambda$ can be decomposed into $\mathbb {TOW}_{\omega _1}*(\mathbb {TOW}_\lambda /\mathbb {TOW}_{\omega _1})$. By Sect. 3.2, $\mathbb {TOW}_{\omega _1}$ is equivalent to an iteration of length $\omega _1$ of Mathias forcings with respect to countably generated filters, therefore it is equivalent to the Cohen forcing which adds $\omega _1$ many Cohen reals. Since these $\omega _1$ many Cohen reals form an unbounded family, it follows that $V^{\mathbb {TOW}_{\omega _1}}\models ``{\mathfrak {b}}=\omega _1$”. In $V^{\mathbb {TOW}_{\omega _1}}$, let ${\mathcal {B}}$ be an unbounded family of size $\omega _1$ which is countably directed. The quotient $\mathbb {TOW}_\lambda /\mathbb {TOW}_{\omega _1}$ is equivalent to a finite support iteration of Mathias forcings with respect to filters which are ${\mathcal {B}}$-Canjar (which follows as in the proof of Theorem 3.7), therefore ${\mathcal {B}}$ is unbounded in $V^{\mathbb {TOW}_\lambda }$, thus, using that ${\mathfrak {t}}\le {\mathfrak {b}}$, we get the following:

### Corollary 5.1

Let $\lambda$ be a regular uncountable cardinal. Then the following holds in $V^{\mathbb {TOW}_\lambda }$:

1. $${\mathfrak {t}}={\mathfrak {b}}=\omega _1$$.
2. There exist towers^4^ of length $\omega _1$ and of length $\lambda$.
3. There exist unbounded scales of length $\omega _1$ and of length ${\mathfrak {b}}^{V}$ (and of any length $\kappa$ for which there exists an unbounded scale in the ground model *V*).

The analogous argument works for $\mathbb {MAD}_\lambda$, so we get the following:

### Corollary 5.2

Let $\lambda$ be a regular uncountable cardinal. Then the following holds in $V^{\mathbb {MAD}_\lambda }$:

1. $${\mathfrak {t}}={\mathfrak {b}}=\omega _1$$.
2. There exists^5^ a mad family of size $\lambda$.
3. There exist unbounded scales of length $\omega _1$ and of length ${\mathfrak {b}}^{V}$ (and of any length $\kappa$ for which there exists an unbounded scale in the ground model *V*).

## Questions

Finally, let us list a few questions, which the anonymous referee suggested to add to the paper. Note that $\mathbb {TOW}_\lambda$ and $\mathbb {MAD}_\lambda$ are forcing equivalent in case $\lambda \le \omega _1$, because in this case both can be written (see Sects. 3.2, 4.2) as finite support iterations of Mathias forcings with respect to countably generated filters (which are just Cohen forcing). We strongly conjecture, however, that this is not the case for larger $\lambda$:

### Question 6.1

Are $\mathbb {TOW}_\lambda$ and $\mathbb {MAD}_\lambda$ forcing equivalent for $\lambda > \omega _1$?

The above question could be settled by showing that $\mathbb {MAD}_\lambda$ adds an object which is not added by $\mathbb {TOW}_\lambda$, or vice versa:

### Question 6.2

Let $\lambda > \omega _1$. Does $\mathbb {TOW}_\lambda$ add a mad family of size $\lambda$? Does $\mathbb {MAD}_\lambda$ add a tower of length $\lambda$?

For regular uncountable $\lambda$, both $\mathbb {TOW}_\lambda$ and $\mathbb {MAD}_\lambda$ force ${\mathfrak {t}}= {\mathfrak {b}}= \omega _1$ (see Corollary 5.1 and Corollary 5.2).

### Question 6.3

Does $\mathbb {TOW}_\lambda$ force ${\mathfrak {a}}= \omega _1$? Does $\mathbb {MAD}_\lambda$ force ${\mathfrak {a}}= \omega _1$?

## Footnotes

1 In fact, ${\mathbb {C}}$ is almost bounding.

2 In fact, [9, Theorem 2.2] is a much more general version than the one presented here.

3 This technical part of the genericity argument is easier than in case of the tower forcing, because it is enough to extend the *s*’s by 0’s. In the case of tower forcing, on the other hand, we have to add 1’s, which needs some preparation.

4 The generic object added by $\mathbb {TOW}_{\omega _1}$ is a tower of length $\omega _1$ in $V^{\mathbb {TOW}_{\omega _1}}$, but it is clearly not a tower in $V^{\mathbb {TOW}_\lambda }$ any more.

5 Of course, there also exists a tower of length $\omega _1$, as in $V^{\mathbb {TOW}_\lambda }$.
